# Mycetoma in the Sudan: An Update from the Mycetoma Research Centre, University of Khartoum, Sudan

**DOI:** 10.1371/journal.pntd.0003679

**Published:** 2015-03-27

**Authors:** Ahmed Fahal, EL Sheikh Mahgoub, Ahmed M. EL Hassan, Manar Elsheikh Abdel-Rahman

**Affiliations:** 1 Mycetoma Research Centre, University of Khartoum, Khartoum, Sudan; 2 Faculty of Mathematical Sciences, University of Khartoum, Khartoum, Sudan; Fundação Oswaldo Cruz, BRAZIL

## Abstract

This communication reports on the Mycetoma Research Centre of the University of Khartoum, Sudan experience on 6,792 patients seen during the period 1991–2014.The patients were predominately young (64% under 30 years old) males (76%). The majority (68%) were from the Sudan mycetoma belt and 28% were students. *Madurella mycetomatis* eumycetoma was the most common type (70%). In 66% of the patients the duration of the disease was less than five years, and 81% gave a history of sinuses discharging mostly black grains (78%). History of trauma at the mycetoma site was reported in 20%. Local pain was reported in 27% of the patients, and only 12% had a family history of mycetoma. The study showed that 57% of the patients had previous surgical excisions and recurrence, and only 4% received previous medical treatment for mycetoma. Other concomitant medical diseases were reported in 4% of the patients. The foot (76%) and hand (8%) were the most commonly affected sites. Less frequently affected sites were the leg and knee (7%), thigh (2%), buttock (2%) and arm and forearm (1%). Rare sites included the chest wall, head and neck, back, abdominal wall, perineum, oral cavity, tongue and eye. Multiple sites mycetoma was recorded in 135 (2%) of cases. At presentation, 37% of patients had massive lesions, 79% had sinuses, 8% had local hyper-hydrosis at the mycetoma lesion, 11% had regional lymphadenopathy, while 6% had dilated tortuous veins proximal to the mycetoma lesions. The diagnosis of mycetoma was established by combined imaging techniques and cytological, histopathological, serological tests and grain culture. Patients with actinomycetoma received a combination of antimicrobial agents, while eumycetoma patients received antifungal agents combined with various surgical excisions. Surgical excisions in the form of wide local excision, debridement or amputation were done in 807 patients, and of them 248 patients (30.7%) had postoperative recurrence. Different types of amputations were done in 120 patients (1.7%).

## Author Summary

Many researchers consider the Sudan as the mycetoma homeland. The first report on mycetoma was at the turn of the eighteenth century, and since then many documents on mycetoma have been reported. However, there is no recent report on mycetoma in the country. In 1977 Mahgoub published data on mycetoma in Sudan, but no more data were published until the MRC was established. The present study reports on 6,792 patients with mycetoma seen and managed at the MRC in a period of 23 years. This is the largest reported number of cases on the disease at national and international levels. The clinical presentations of the reported patients were in line with the previous reports on mycetoma from the Sudan and elsewhere. Still, many patients presented late with advanced disease and enormous disabilities and deformities. Some of them had a fatal outcome due to several complications. Despite advances in the diagnosis and treatment of this disease, outcome is still unsatisfactory. There is a need for more research to develop effective treatment of mycetoma and field friendly diagnostic techniques. Adequate preventive and control measures to reduce the disease morbidity and mortality are needed.

## Introduction

Mycetoma is a unique neglected tropical disease. It is a morbid chronic progressive inflammatory condition caused by certain fungi (eumycetoma) or bacteria (actinomycetoma) [1, 2, 3]. It is characterised by devastating distortions, disabilities, high morbidity and it has various negative impacts in term of health and socio-economically on patients, communities and health authorities [4, 5]. Young adult patients are affected most but no age is immune [6, 7]. It is more frequently reported in farmers, shepherds and workers of low socio-economic status [8, 9]. The triad of a painless subcutaneous mass, multiple sinuses and purulent or sero-purulent discharge that contains grains is pathognomonic of mycetoma [10, 11, 12]. The mass usually spread to involve the skin and the deep structures, resulting in destruction, deformity and loss of function, and occasionally it can be fatal. The limbs are the most frequently affected sites and that is seen in more than 80% of patients [13]. Mycetoma patients tent to present late with massive disease due to multifactorial factors [14].

The current diagnostic tools for mycetoma are varied and that include imaging, molecular and histopathological techniques, sero-diagnostic tests, as well as the classical grain culture. It is interesting to note, most of these investigations are not available in mycetoma endemic area [15, 16, 17, 18, 19].

Presently, it is quiet perplexing and challenging to treat patients with mycetoma and the treatment outcome is unsatisfactory. It is characterized by low cure rate and high amputation and high recurrence rates. Currently there is no control or prevention programmes for mycetoma [20, 21].

In an attempt to bridge the knowledge gap in mycetoma, the Mycetoma Research Centre (MRC) was established in 1991 under the auspices of the University of Khartoum and based at Soba University Hospital. It is the only referral centre in the country which aims to provide integrated high quality medical care for mycetoma patients, education and training for medical and health professionals, community development activities and to lead in mycetoma research. In this communication, we report on the MRC 23 years’ experience in managing mycetoma patients.

## Materials and Methods

This descriptive, cross-sectional hospital based study was conducted at the Mycetoma Research Centre, University of Khartoum, Khartoum, Sudan. The study included 6,792 patients with confirmed mycetoma seen in the period January 1991 and July 2014. The diagnosis of mycetoma was confirmed by careful interview, meticulous clinical examinations and certain investigations.

The investigations included fine needle aspiration for cytology (FNA) and histopathological examination of surgical biopsies. In the latter, different staining techniques were used. The commonest was haematoxylin and eosin (H&E) stain which was adequate for identifying most of the grains. When the grains identification was not conclusive, special stains were used and that included periodic acid-Schiff (PAS) and methenamine silver stains.

Culture of the grain is still the most important method for the diagnosis of mycetoma. Grains obtained from surgical biopsies were inoculated in different media. Eumycetes grains were cultured in blood agar and Sabouraud dextrose agar and were incubated at 37°C for six to eight weeks. Actinomycetes grains were cultured on blood agar, brain heart infusion or Lowenstein- Jensen medium and incubated at 37°C for 3 to 4 weeks.

The sero-diagnostic test employed was counter-immuno-electrophoresis as described previously [22]. Various imaging techniques were used which included plain radiography of the affected parts in at least two views; anterio-posterior and lateral, lesion ultrasound examination was also used. In selected patients with advanced disease, MRI and CT scan were performed.

### Statistical analysis

Statistical analysis was conducted using Stata 12. Data was summarized as percentages for categorical variables and mean ± standard error of the mean (SEM) and median for continuous variables.

### Ethics statement

An ethical clearance was obtained from Soba University Hospital Ethical Committee to conduct the study. As this study is a retrospective one, done by reviewing patients’ notes the Ethical Committee waived the patients’ informed consents. All medical records were anonymized

## Results

### Epidemiology and clinical presentation

The study included 6,792 patients with confirmed mycetoma; 5,150 (76%) of them were males and 1,642 (24%) were females. Their age ranged between 3 and 88 years with median of 25 years (mean 29 ± 0.2 standard error). Most of them, 4,353 (64%), were less than 30 years old at presentation, 1,586 (23%) were under 20 years old and 2,435 (36%) were 30 years old or more (Table 1).

**Table 1:** The demographic characteristics of the studied population.

Demographic Characteristics	No.	%
**Sex**		
Male	5,150	75.8
Female	1,642	24.2
**Age in years**		
<20	1,586	23.4
20–39	2,767	40.7
30+	2,435	35.9
Missing	4	0.1
**Occupation**		
Student	1,872	27.6
Worker	1,239	18.2
Farmer	1,239	18.2
Jobless	628	9.2
Employee	104	1.5
Clerk	69	1.0
Others	730	10.7
House wife	882	13.0
Missing	29	0.4
**Duration**		
<1	1,691	24.9
>1–5	2,798	41.2
>5–10	1,330	19.6
>10–20	701	10.3
>20–30	163	2.4
30+	52	0.8
Missing	57	0.8
**Grains**		
None	3,824	56.3
Present	2,830	41.7
Missing	138	2.0
**Discharge**		
Yes	5,465	80.5
No	919	13.5
Unknown	372	5.5
Missing	36	0.5
**Pain**		
Yes	1,842	27.1
No	4,790	70.5
Sometimes	32	0.5
Missing	128	1.9
**Trauma**		
Yes	1,367	20.1
No	4,586	67.5
Not sure	774	11.4
Missing	65	1.0
**Family history**		
Yes	811	11.9
No	5,127	75.5
Missing	854	12.6
**Other Medical problem**		
Yes	260	3.8
No	6,509	95.8
Missing	23	0.3
**Number of previous surgeries**		
0	2,944	43.4
1	2,545	37.5
2	789	11.6
3	296	4.4
4	205	3.0
Missing	12	0.2
Total	6,791	100.0

In this study 1,872 (28%) students were affected. This was followed by farmers 1,239 (18%) and manual domestic workers 1,239 (18%). Due to the prolonged illness and disability, 628 (9%) patients were unemployed. Housewives constituted 13% of the patients (Table 1).

The study showed that, 2,476 patients (37%) were from Gezira State; 837 patients (12%) were from White Nile State and 747 patients (11%) were from North Kordofan State. There was also a significant number of patients from the Capital, Khartoum State 1,037 (15%) (S1 Fig). Darfur States were the least affected area. In this series, thirty three patients were from neighbouring countries of Chad, Ethiopia, Saudi Arabia, Eritrea and some were from Yemen.

The duration of the disease at presentation ranged between few months and 60 years with a median duration of 3 years (mean 6 ± 0.1 standard error). The majority of the patients 5,819 (86%) had mycetoma for 10 years or less; 1,691(24%) had the infection for less than one year. Only 51(1%) patients had the disease for more than 30 years, (Table 1).

The majority of patients, 5,465 (81%), gave a history of discharge that contained grains (Table 1) and the commonest were black grains (78%). Eleven percent of the grains were yellow, eight percent were white and two percent were red. In this series, about a quarter of patients 1,842 (27%) had painful lesions (Table 1).

Local trauma at the mycetoma site was recalled by 1,367(20%) of the patients, while the majority of patients 4,586 (68%) had no recollection of local trauma and 774(11%) patients were not certain. Different types of trauma were mentioned and that included thorns pricks, stones cuts, trauma in framing and football games and snake bites. Most of the traumas were considered minor.

Only 260 (4%) patients had concomitant medical problems that included diabetes (n = 33), hypertension (n = 22), tuberculosis (n = 9), leprosy (n = 3), renal diseases and renal transplant (n = 7) and others. Family history of mycetoma among the study population was documented in 811 (12%) of patients (Table 1).

Over half of the patients 3,847 (57%) had previous surgical excisions performed elsewhere and the number of excisions ranged between one to four excisions, The surgery was performed under general anaesthesia in 1,746 (50%), 1,199 (34%) had local anaesthesia and the rest had spinal anaesthesia. Only 194 (4%) of the patients had previous medical treatment for the mycetoma (Table 1).

**Fig 1:**
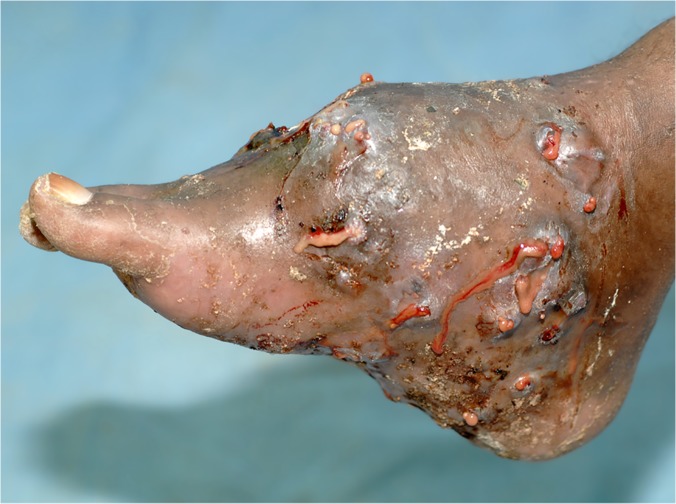
Showing massive foot eumycetoma with multiple sinuses and discharge with black grains.

**Fig 2:**
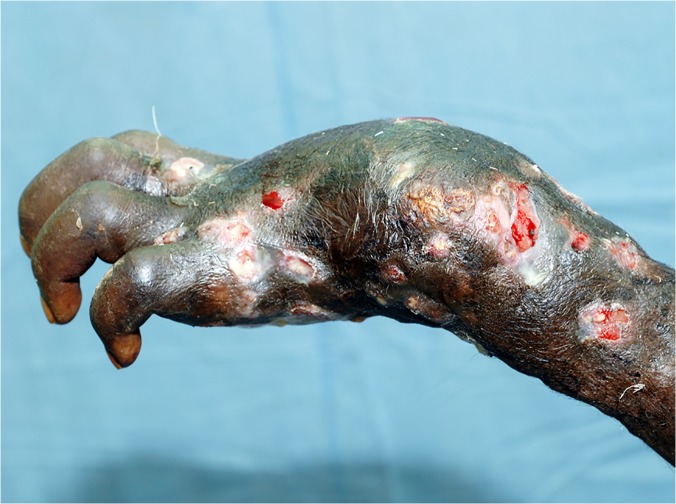
Showing massive hand and forearm eumycetoma with massive deformity.

**Fig 3:**
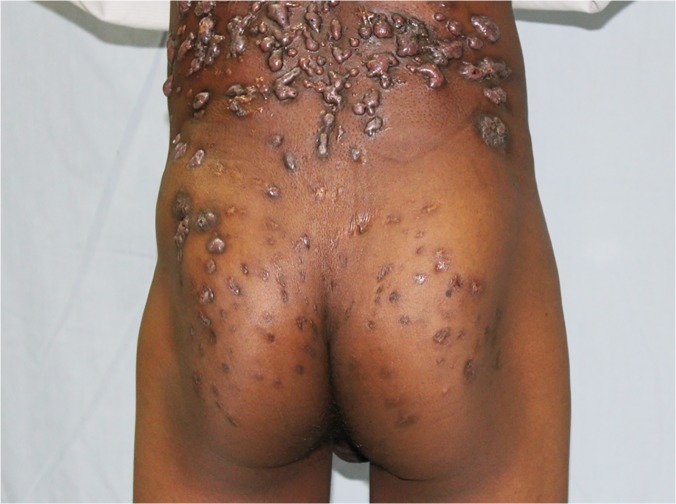
Showing massive back and gluteal eumycetoma.

**Fig 4:**
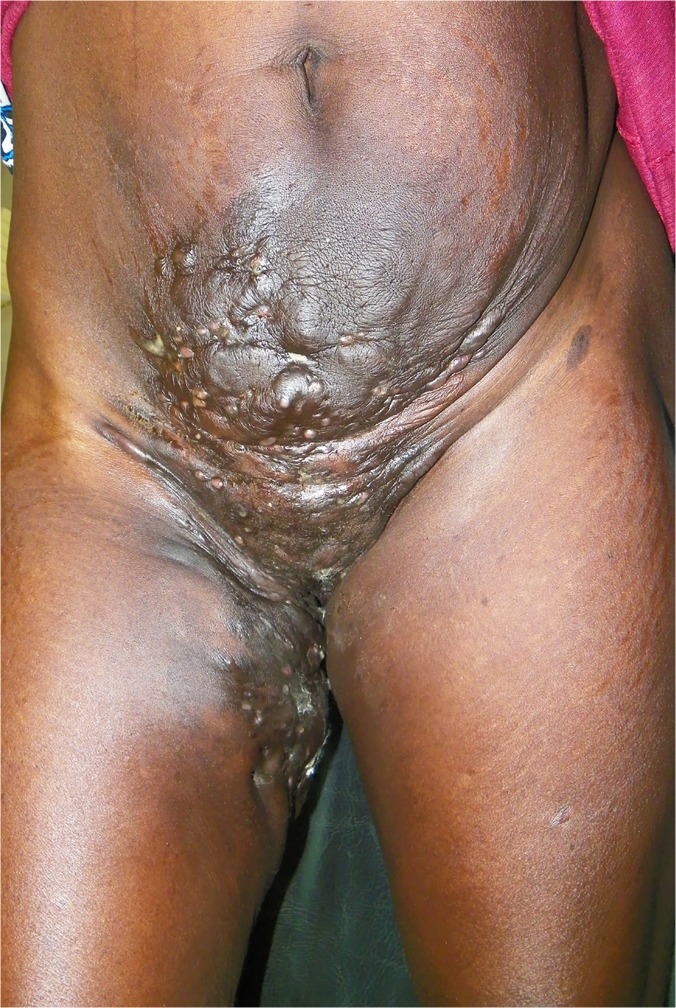
Showing massive anterior abdominal wall, perineal, vulval and upper thigh actinomycetoma

In this study, the foot was the most common site for mycetoma and it occurred in 5,151 (75.8%) patients. The right and left foot were affected in 2,239 (33%) 2,176 (32%), respectively. Thus both feet were affected to the same extent. The hand was the second most common site for mycetoma with a frequency of 507(7.5%). The right hand was affected most and it was documented in 307 patients (5%), the affection of the right hand is significant statistically, (Table 2, Figs. 1, 2). Less frequently affected sites were the leg and knee (7%), thigh (2%), buttock (2%) and arm & forearm (1%). Few patients had mycetoma in the chest wall, head and neck, back, abdominal wall or perineum (Figs. 3, 4).

**Table 2:** The mycetoma sites among the studied population.

Site	No.	%
Foot	5,151	75.8
Hand	507	7.5
leg & knee	454	6.7
Thigh	112	1.6
Buttock	103	1.5
Arm & forearm	77	1.1
Head & neck	53	0.8
Back	29	0.4
Perineum	18	0.3
Chest wall	09	0.1
Abdominal wall	09	0.1
Others	70	1.0
Multiple	135	2.0
Both feet	06	0.1
Missing	59	0.9
**Total**	6,792	100.0

In this series, 135 patients (2%) had multiple mycetomata affecting different body sites. These were primary lesions not due to lymphatic spread. The causatives organisms were the same in 90% of them while in 10%, the isolated organisms from the different mycetoma sites were different. The foot was the common site in this group of patients, (Table 2).

The mycetoma lesions were classified according to their size into small lesion (less than 5 cm in diameter), moderate lesion (5–10 cm) and massive lesion (>10 cm). The study showed that 2,504 (37%) patients had massive lesion at presentation while 2,095 (31%) patients had small lesions. At presentation, sinuses were observed in 5,343 (79%) and among these 2,828 (53%) were active discharging grains while in 1,501 (28%) the sinuses were healed while 1,014 (19%) patients had both active and healed sinuses (Table 3).

**Table 3:** The clinical findings among the study population.

Clinical Findings	No.	%
**Size**		
Less than 5cm	2,095	30.8
5-10cm	1,775	26.1
More > 10cm	2,504	36.9
Operated	192	2.8
Missing	226	3.3
**Sinuses**		
No	1,366	20.1
Yes	5,343	78.7
Operated	21	0.3
Missing	62	0.9
**Type of sinuses**		
Active	2,828	52.9
Healed	1,501	28.1
Both	1,014	19.0
**Veins**		
None	6,122	90.1
Present	422	6.2
Missing	248	3.7
**Sweating**		
None	5,623	82.8
Present	532	7.8
Missing	637	9.4
**Lymphadenopathy**		
None	5,891	86.7
Present	699	10.3
Missing	202	3.0

Local hyper-hydrosis at and around the mycetoma lesion was detected in 514 (7.9%) patients. Regional lymphadenopathy was detected in 699 (11%) patients. Dilated tortuous veins proximal to the mycetoma lesions were observed in 422 (6%) patients, (Table 3).

### Diagnosis

**Fig 5:**
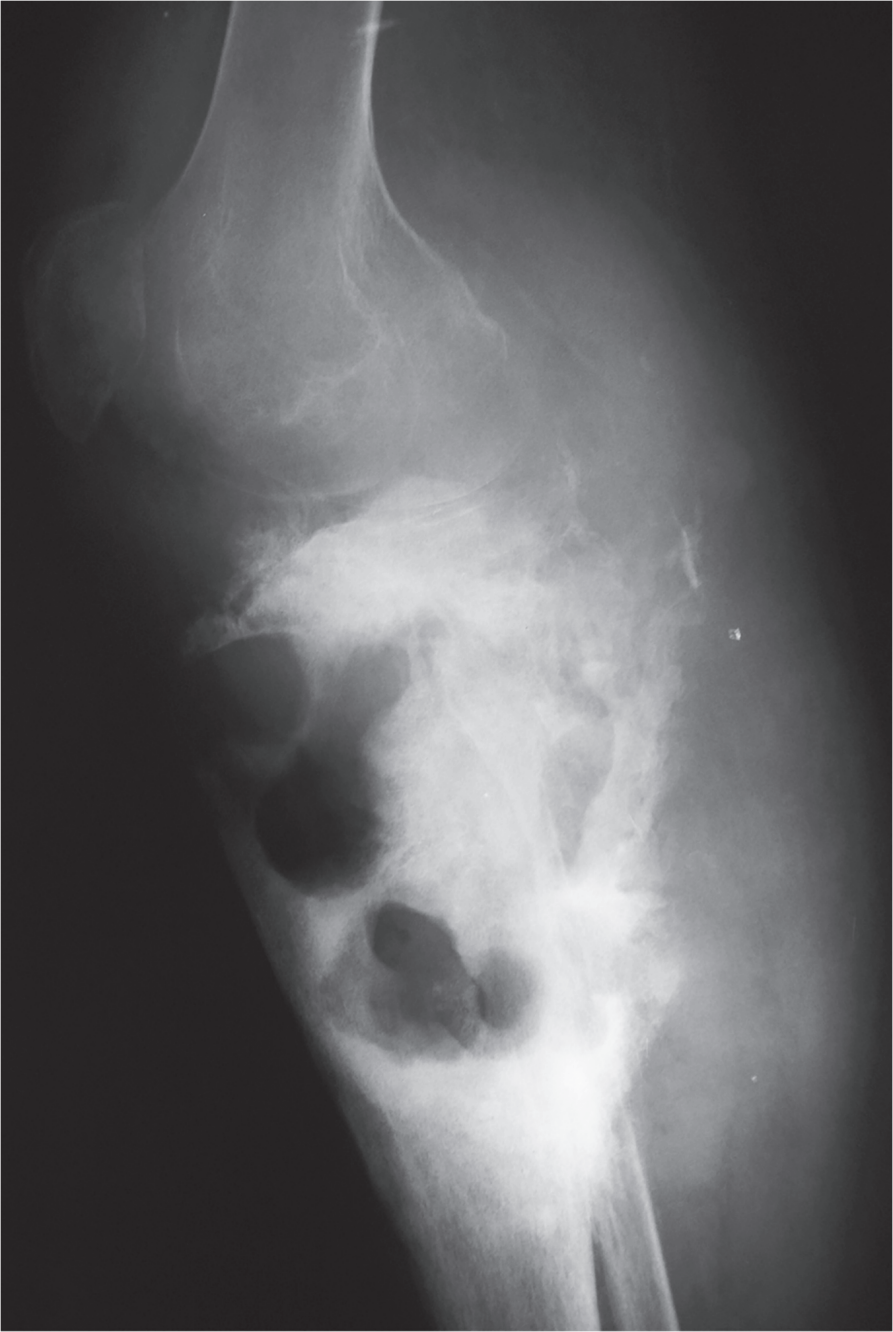
Showing knee region X-ray with type finding of eumycetoma; soft tissue mass, periosteal reaction of the lower part of the femur, patella and upper tibia and multiple bone cavities in the upper part of the tibia.

X-Ray examination of the affected sites was performed in 4,508 (66%) patients. It was normal in 1,235 (27%). A soft tissue mass was seen in 1,709 (38%), bone destruction was detected in 794 (17%), a periosteal reaction was found in 180 (4%) and in 590 (13%) a combination of these findings were detected, (Fig. 5).

In the early days of the MRC suspected mycetoma lesions were diagnosed by histopathological examination of surgical biopsies specimens. Recently, we used Fine Needle Aspiration (FNA) from lesions to identify the grains and the tissue reaction. If FNA was negative in a suspicious case surgical biopsies and histopathological examination were done. In the present study, 3,177 (47%) patients had FNA for cytology. The diagnosis was *M*. *mycetomatis* in 2,379 (75%) patients, *Actinomadura madurae* in 316 (10%) patients, *Streptomyces somaliensis* in 277 patients (7%) *Actinomadura pelletieri* was uncommon and was found in 39 (1%) of cases.

**Fig 6:**
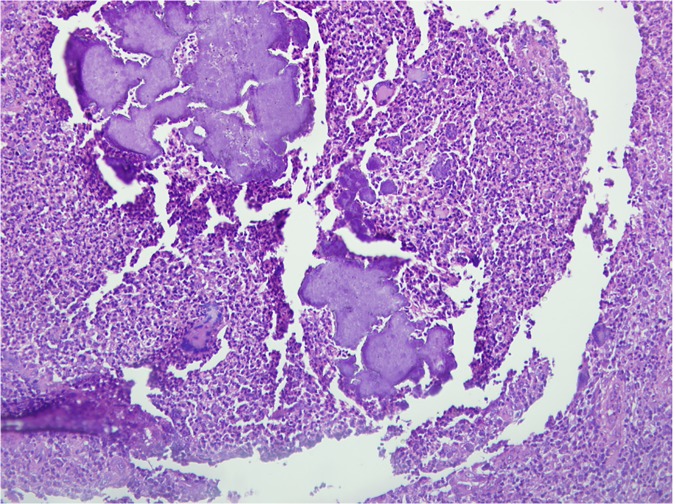
Microphotograph showing many *Actinomadura pelletieri* grains surrounded by multi-inflammatory cells. H&E X 200.

The total number of patients who had surgical biopsies and histopathological examinations was 2,557(38%) patients. Among them, the diagnosis of *M*. *mycetomatis* was established in 1,714 (67%) patients, *Streptomyces somaliensis* in 517 (20%) patients, *Actinomadura madurae* in 140 (6%) patients, *Actinomadura pelletieri* in 45 (2%) and in eight patients (0.3%) other organisms were identified, (Fig. 6).

**Fig 7:**
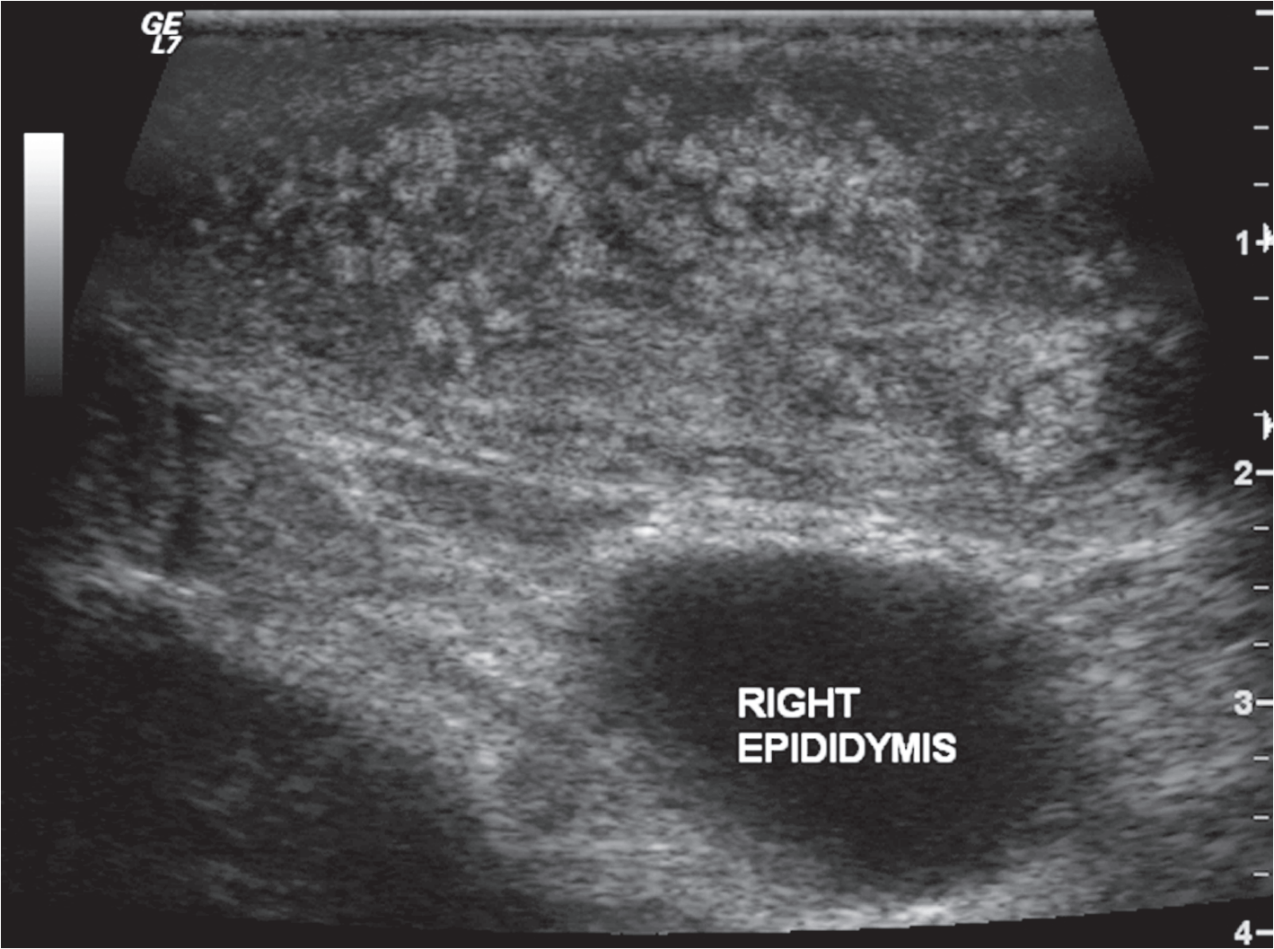
Showing the ultrasound appearance of eumycetoma lesion with numerous grains producing numerous and sharp hyper-reflective echoes. There are multiple thick-walled cavities with no acoustic enhancement.

Ultrasound examination of the mycetoma lesion was performed in 2,204 (33%) patients. Eumycetoma was diagnosed in 1,599 (73%) patients, actinomycetoma in 299 (14%) while in 291(13%) no diagnosis was established, (Fig. 7).

**Fig 8:**
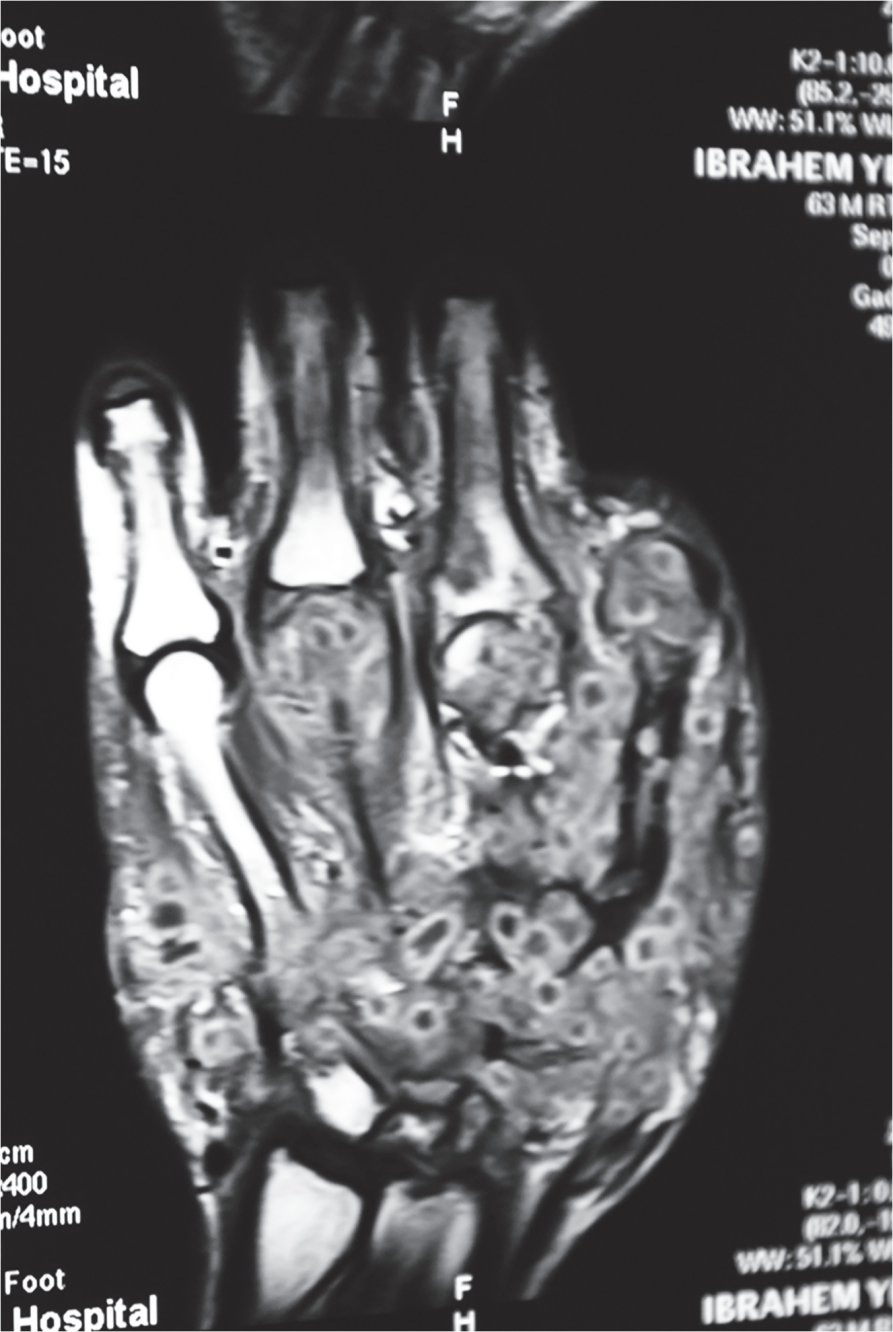
Showing hand MRI with an extensive eumycetoma lesion infiltrating and obliterating the subcutaneous tissue planes. There are multiple low signal intensity fungal grains, bone marrow oedema, and a dot-in-circle sign.

MRI was performed in 102 patients, it showed different signs and that included the infiltration of the skin, subcutaneous tissues, muscles and bones with mycetoma grains and inflammatory tissue, obliterating the subcutaneous tissue planes and the dot-in-circle sign which is characteristic of mycetoma, (Fig. 8).

The final diagnosis in this series based on the various diagnostic tools was eumycetoma in 4,754 (70%) patients and the common organism was *Madurella mycetomati*s. Actinomycetoma was diagnosed in 2038 (30%) patients and common causative organisms were *Streptomyces somaliensis*, *Actinomadura madurae*, and *Actinomadura pelletieri*.

Using different molecular techniques, three rare causative organisms were identified and these were *Streptomyces sudanensis* a novel causative agent for actinomycetoma, *Pleurostomophoraochracea*, another novel agent of human eumycetoma with yellow grains and *Madurella fahalii* an uncommon eumycetoma agent [23, 24, 25].

### Treatment

Patients were treated according to the type of mycetoma. For actinomycetoma a combination of antimicrobial agents was given. In the past, the combination of choice was streptomycin sulphate and dapsone. If there was no response combination of streptomycin and trimethoprim-sulfamethoxazole was given and treatment duration ranged between 6 months and four years with a mean of 18 months. More recently, trimethoprim-sulfamethoxazole at a dose of 8/40 mg/kg/day for 5 weeks and amikacin at 15 mg/kg/day in a divided dose every 12 hours were given for 3 weeks and these drugs were given in the form of cycles. The cycles number ranged between 5 to ten cycles. The renal and audiometric functions are monitored closely during the treatment [20].

For eumycetoma several antifungal agents were used combined with various forms of surgical excisions and the later included wide local excision, debridement and amputation. Oral 400–800 mg/day Ketoconazole was the drug of choice as recommended by Mahgoub & Gumaa in 1984 [26]. However, it was recently banned due to its high toxicity and side effects. Recently, 200–400 mg/day Itraconazole is used as the drug of choice with less toxicity and side effects [27]. The duration of treatment ranged between six months and 3 years.

Surgical excisions in the form of wide local excision, debridement or amputation were done in 807 patients and of them 248 patients (30.7%) had postoperative recurrence. Different types of amputations were done in 120 patients (1.7%).

## Discussion

The history of mycetoma in the Sudan is long and exciting. Slede and colleagues reported on 1729 hospital patients with mycetoma from different parts of Sudan seen in the period 1951–1952 [28]. From the hospitals records, Abbot in 1956 reported 1231 patients seen over a period of 30 months [29]. In 1964, Lynch estimated the incidence of mycetoma in the Sudan as 300–400 patients per year [30]. From Central Sudan, Moghraby reported on 817 mycetoma patients seen at Wad Madani Civil Hospital, Central Sudan, in a period of 11 years [31]. Mahgoub from the Mycetoma Clinic at Khartoum North and the Ministry of Health records, reported an incidence of 365 new patients annually seen in the period between 1971–1975 [32]. Following these early reports and for various reasons, there were no reports on the disease in the Sudan. With this background this study was carried out to bridge the knowledge gap in mycetoma in the Sudan. The study reports on the largest number and types of mycetoma and their geographic distribution ever recorded nationally or internationally.

The annual incidence of newly diagnosed patients with mycetoma at the MRC is 355 and this is in line with that reported previously by Mahgoub [32]. However, the prevalence of mycetoma in one endemic village in the While Nile State, Central Sudan was recently found to be 14.5 /1000 inhabitants [33]. The incidence reported in this communication may be the tip of the iceberg representing only the few patients who were able to reach the MRC seeking medical care.

Generally, mycetoma patients tend to present at a late stage with advanced disease as reported in this study. This may be explained by the substantial lack of health education among communities and patients in endemic regions, scarcity of medical and health facilities in rural endemic areas and the patients’ low socio-economic status. To overcome this, vigorous health education programmes need to be introduced in mycetoma endemic areas.

Male predominance in mycetoma reported in this series is in accordance with all previously reported series form the Sudan [28–32].This cannot be explained solely on outdoor activities since in parts of Sudan where mycetoma is endemic, females are also committed to these activities. This is supported by recent field study which showed both sexes were equally affected [33]. However, genetics and hormonal factors may explain this male predominance and further in-depth studies are needed.

This study is in agreement with the medical literature which showed that young population is affected most [28–32].The explanation of this finding is unclear; the early traumatic exposure to the causative organisms in the soil during playing or field activities may offer an explanation. All the reported series showed cultivators, shepherd and field workers are the frequent affected groups [28–32, 34, 35]. It is interesting to note that, students were affected most in this study. The explanation of this finding is an enigma. However it is known in endemic areas, students usually help their families during their outdoor activities but further studies should address this issue.

As the mycetoma belt passes across Gezira, Sinnar, While Nile and Kordofan States, and they have similar geographical and environmental characteristics, the majority of the study population were from these states and this is in accordance with previous reports [28–32]. It is interesting to note, in this study, a considerable number of the patients were from Khartoum State, which is a non-endemic area. This may be explained by the massive migration of tribes from the endemic areas to Khartoum due to numerous socio-economic reasons. Some of those migrants often go back to visit their homelands and they may get infected and then return to Khartoum. This problem needs be addressed and clarified. Due to the steady increase in international movements and travel some of the patients from the neighbouring countries of Chad, Ethiopia, Yemen, Saudi Arabia and Eritrea were seen and treated at the MRC.

Most of the patients had a long standing disease at presentation. This is in line with most of the previously reported series [28–32, 34, 35]. This has been attributed to lack of health education, patients’ poor socio-economic status and the absence of medical and health facilities at the endemic regions. To address these short comings, MRC conducted several health campaigns at endemic areas where health education activities were conducted and surgical treatment was performed by mobile surgical unit [33]. The MRC has even established wards and a small laboratory in the White Nile State.

The clinical trial of the subcutaneous mass, multiple sinuses and discharge that contained grains reported in this series is typical of mycetoma and it is in line with previous reports [25–29, 31, 32]. Although mycetoma can be diagnosed clinically, yet this is not conclusive, hence, accurate and explicit diagnostic tools are required to advise the proper treatment.

Typically mycetoma is a painless condition and this is an important cause for the delayed presentation in the majority of patients. In this communication, only 27% of the patients had local pain at presentation. The pain typically presents in bouts due to secondary bacterial infection in the lesion and for various socio-economic and cultural reasons patients tolerate these bouts.

**Fig 9:**
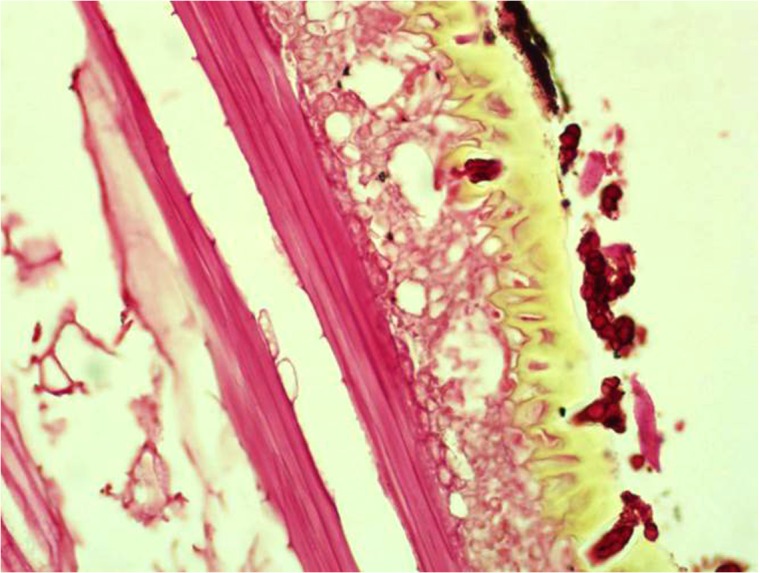
Showing a thorn with vesicular structures and a black pigment of *M. mycetomatis* removed surgically from one patient (H&EX200).

It is widely believed that, the practice of walking barefooted and working in the field barehanded predispose to traumatic inoculation of the causative organisms. However, in this study, only 20% of the patients had recalled history of local trauma but they might have had minor unnoticed trauma that facilitated the organisms’ inoculation. However, if the traumatic inoculation theory is true then the incidence of mycetoma should be much higher than it is. However, on the other hand, the presence of thorns within the mycetoma lesions during surgery and fungal hyphae within the thorns may favour the traumatic inoculation theory (Fig. 9) but this may be co-incident finding. Further detailed community based studies are warranted to clarify and offer an explanation.

It is interesting to note, that the isolation of *Madurella mycetomatis*, from the soil was not possible despite detection of its DNA in the soil [36] and also it is known that, the production of mycetoma granulomas by inoculation in experimental animals is difficult [37] all these may favour the theory of the presence of an intermediate host for mycetoma development.

In this large series, most of the patients had no concomitant medical conditions that predispose to the infection. Sera from 100 patients with mycetoma were tested for HIV and were negative and clinical evidence of HIV was not encountered in this study. In this series, few patients had tuberculosis and leprosy with mycetoma but it is difficult to ascertain whether they were the predisposing factor for mycetoma or vice versa [38]. Seven patients were on immune-suppressive therapy for renal transplant and it seems that they had re-activation of old mycetoma lesion as all of them had past history of surgically treated disease.

Family history of mycetoma among the study population was documented in a minority of patients (12%). This low incidence has no clear explanation as in endemic areas, all the population are at risk of contracting the infection because they share the same epidemiological risk factors. We cannot rule out that, some individuals may be immune to mycetoma as a result of healed small inoculations or some individuals may be naturally resistant to the infection. Further epidemiological, immunological and genetic studies are needed to explain these observations.

Surgical recurrence in mycetoma is a common and serious problem that leads to major morbidity and disability. In this study, most of the patients had past history of recurrent disease with repeated surgical excisions performed elsewhere before presentation to the MRC with massive distortions and mutilation. The explanation of the surgical recurrence is multifactorial. Many patients present late with advanced disease and the excisions were incomplete. Furthermore surgery is often performed by inexperienced junior medical doctors or health assistants in the rural areas under local anaesthesia. It is unlikely that the whole lesion is completely removed under these conditions. Mycetoma commonly spread widely along the fascial planes, lymphatics and blood [39] in the form of hyphae and grains and the latter proved to have numerous protective mechanisms [40].

In this study, the foot and hand were the commonly affected sites which is in agreement with the medical literature [28–32, 34, 35]. Rare mycetoma sites in this study included the eye, scrotum, oral cavity, palate and tongue. This has been observed by others [41–44].

Extra pedal mycetoma was encountered in the studied population but less frequent, which is again in agreement with other reports [41–44]. It is interesting to note, in the different sites, eumycetoma was the commonest type which contradicts some other reports which showed most of the extra-pedal mycetoma are actinomycetoma [34, 35]. This may be explained by the fact that *M*. *mycetomatis* is the commonest causative organism in the Sudan

The clinical presentation of multiple mycetomata that were not in the same lymphatic drainage line was encountered in this series. Patients had presented with massive advanced multiple lesions which posed a management challenge. The explanation of this phenomenon is unclear; though, double inoculation of the causative organism is the popular theory.

Most of the patients presented with long standing disease and massive lesions. However, it is interesting to note, in a recently study in an endemic village in Central Sudan, many patients presented with long standing small lesions and the diagnosis of mycetoma was a surgical surprise [33]. The explanation of this presentation is unclear. However the repeated exposure to the subclinical infection may have stimulated the immune system to localise the infection.

In this communication, the disease onset, course and progress were typical and it is characterised by high morbidity and disability in particularly with late advanced disease. In some patients, the disease has progressed wildly and aggressively involving the deep organs such as the urinary bladder, pelvic organs and bones, spinal cord, lung and other structures and some infections resulted in fatal outcome which is uncommon in mycetoma [45–51].Further immunological and genetic studies are recommended to explain this aggressive behaviour in some patients.

The diagnosis of mycetoma in this series was based on the combination of clinical and various laboratory and imaging techniques. Proper diagnosis is essential for advice the appropriate treatment. It is essential to note, most of these diagnostic tools are not available in endemic regions. They are also invasive and expensive to both patients and health authorities. This necessitates the need to have a simple, field friendly and affordable diagnostic tools for mycetoma [19].

In this series, the actinomycetoma was less prevailed than eumycetoma. Most of the causative organisms were diagnosed as *Streptomyces somaliensis* but recently with the use of PCR, new organism; *S*. *sudaniensis was* diagnosed which was previously diagnosed as *S*. *somaliensis* by H&E stains [52]. *Actinomadura madurae*, *Actinomadura pelletieri* and *Nocardia brasiliensis* were rare causes of the disease. This is not in accordance with reports from West Africa and Mexico and this may be explained by geographic and environmental factors [34, 35, 40, 43].

The current treatment of mycetoma as seen in this study was suboptimal and in line with previous reports [23, 53]. Hence there is an urgent need for novel, efficient and cost effective treatment.

In conclusion, in this communication we report on the largest mycetoma population ever reported and the findings are almost in line with that reported previously from the Sudan and elsewhere. The findings of this study indicate the need for more efforts to be done for better management of the mycetoma. In view of the difficulty in the diagnosis and treatment of mycetoma [19, 53], novel diagnostic tools and treatment are desperately needed to reduce the enormous deformity, disability and the high morbidity encountered in most mycetoma patients. The knowledge gap in mycetoma is substantial [54] and this should be reduced for better disease understanding, management and control.

## Supporting Information

S1 Checklist STROBE checklist. (DOC)

S1 Fig Map showing the geographic distribution of the studied population and the mycetoma belt in the Sudan. (PNG)
