# Supplementary figures and images for: Mycetoma in the Sudan: An Update from the Mycetoma Research Centre, University of Khartoum, Sudan
Source: PLoS Negl Trop Dis. 2015 Mar 27;9(3):e0003679. doi: 10.1371/journal.pntd.0003679 (PMC4376889; doi:10.1371/journal.pntd.0003679)

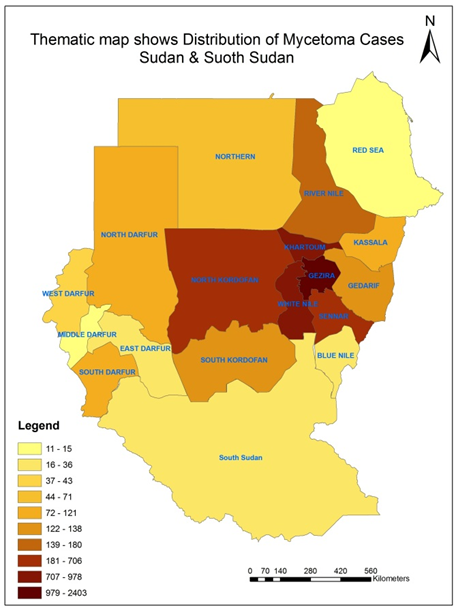

Supplement: S1 Fig — (PNG) [file pntd.0003679.s002.png]
